# Intravenous Laser Blood Irradiation, Interstitial Laser Acupuncture, and Electroacupuncture in an Animal Experimental Setting: Preliminary Results from Heart Rate Variability and Electrocorticographic Recordings

**DOI:** 10.1155/2013/169249

**Published:** 2013-02-11

**Authors:** Wei He, Gerhard Litscher, Xiaoyu Wang, Xianghong Jing, Hong Shi, Hongyan Shang, Bing Zhu

**Affiliations:** ^1^Department of Meridians, Institute of Acupuncture and Moxibustion, China Academy of Chinese Medical Sciences, Beijing 100700, China; ^2^Stronach Research Unit for Complementary and Integrative Laser Medicine, Research Unit of Biomedical Engineering in Anesthesia and Intensive Care Medicine, TCM Research Center Graz, Medical University of Graz, 8036 Graz, Austria

## Abstract

This is the first study to investigate intravenous (i.v.) laser blood irradiation, interstitial (i.st.) laser acupuncture, and electroacupuncture (EA) in combination with heart rate variability (HRV) and electrocorticogram. We investigated 10 male anesthetized Sprague-Dawley rats under the three conditions mentioned previously in Beijing, China, and data analysis was performed in Graz, Europe. For i.v. laser stimulation in the femoral vein and i.st. laser acupuncture at Neiguan (PC6), we used a European system (Modulas needle, Schwa-Medico, Germany; 658 nm, 50 mW, continuous wave mode), and for EA at Neiguan, a Chinese system (Hanshi-100A; Nanjing Jisheng Medical Technology Company, China; 15 Hz, 1 mA). HR, HRV, and electrocorticogram were recorded using a biophysical amplifier AVB-10 (Nihon-Kohden, Japan). HR changed significantly during i.st. laser acupuncture stimulation of Neiguan in anesthetized rats. Total HRV increased insignificantly during i.v. and i.st. laser stimulation. The LF/HF ratio showed significant changes only during i.v. laser blood irradiation. Integrated cortical EEG (electrocorticogram) decreased insignificantly during EA and i.v. laser blood irradiation. Further studies concerning dosage-dependent alterations are in progress.

## 1. Introduction

Intravenous (i.v.) laser blood irradiation was accomplished for the first time approximately 25 years ago in the former Soviet Union [1–3]. Laser light was brought directly into the blood stream through a one-way catheter. In vitro tests showed that biological soft laser irradiation of white blood cells caused various positive effects, in particular expression of immunoglobulins, interferons, and interleukins. After the introduction of the new method, several clinical studies were published, showing additional effects on various metabolic pathways [4–6]. 

A new technique, percutaneous interstitial (i.st.) laser therapy (using a sterile catheter), allows penetration of laser light into deeper tissues for successful treatment of, for example, herniated disks or spinal stenosis [7]. With this technique, it is possible to irradiate the inside of damaged joints directly, which can lead to better therapeutic results [7]. Penetration depths of up to 10 cm can be reached. Besides red and infrared lasers, also green and violet (blue) lasers, which are normally absorbed directly by the tissue, can be applied deep in the joints or acupuncture points and develop their positive microcirculatory effects [8, 9]. Furthermore, it offers the option to treat tumors with combined photodynamic therapy [10]. 

In addition to i.v. and i.st. laser therapy, of interest predominantly in Western medicine, we included electroacupuncture (EA) as a treatment modality in our present study. EA is a well-known method which has been used and investigated over the last 25 years [11].

In the present study, we explored, for the first time i.v., i.st. laser acupuncture and EA in anesthetized rats under stable conditions and analyzed the effects on physiological neurovegetative parameters and bioelectrical brain activity. Similar to previous studies [12, 13], the data were recorded in 10 rats in Beijing, China, and the data analysis was performed in Graz, Austria. 

## 2. Animals and Methods

### 2.1. Sprague-Dawley Rats

Ten male healthy Sprague-Dawley rats (weight: 260–300 g) were kept in an animal house maintained at 24 ± 1°C, with a 12-hour light-dark cycle and free access to food and water for seven days before the experiment. The animals were initially anesthetized with an intraperitoneal injection of 10% urethane (1.2 g/kg, Sigma-Aldrich, St. Louis, USA). Additional sodium pentobarbital was administered if necessary to prolong the anesthetic state. Animals were sacrificed by an overdose of anesthetics after the study. The study was approved by the Institutional Animal Care and Use Committee of the China Academy of Chinese Medical Sciences and was in accordance with the National Institutes of Health guidelines. 

### 2.2. Intravenous Laser Irradiation

Under anesthesia, the rat was fixed in supine position. The skin in the right groin was cleaned and sterilized with 2% iodophor. Skin incision was performed along the right groin, and the femoral vein was exposed and separated about 1.0–1.5 cm. The proximal femoral vein was clipped by a bulldog clamp, and the distal femoral vein was ligated. The laser needle was inserted into the femoral vein and fixed. The bulldog clamp was removed, and the skin incision was purse string sutured.

**Figure 1:**
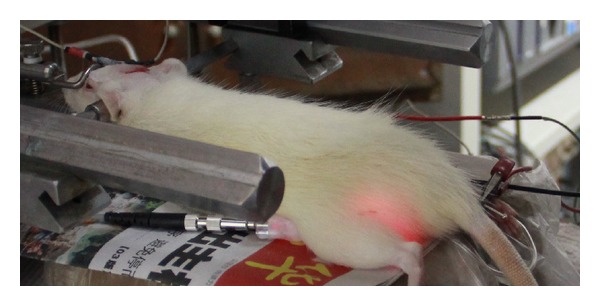
Active i.v. laser blood irradiation.

The laser needle for i.v. laser irradiation (length 35 mm, diameter 0.55 mm) was a Modulas needle (type: IN-Light, Schwa-Medico, Ehringshausen, Germany). It emits red laser light in continuous wave mode with a wavelength of 658 nm and an output power of 50 mW (Figure 1).

### 2.3. Interstitial Laser Acupuncture

Apart from the i.v. irradiation, we stimulated the acupoint Neiguan (PC6) on the left side using i.st. laser acupuncture, using the same type of equipment as for the intravenous irradiation. The laser needle was inserted about 3 mm in the acupoint Neiguan. This acupoint is located proximal to the accessory carpal pad of the forelimb, between the flexor carpi radialis and palmaris longus ligaments. 

### 2.4. Electroacupuncture

Electroacupuncture was also performed at the left Neiguan acupoint. One needle was inserted about 3 mm into the acupoint, and the other needle was inserted into a point 2 mm from the acupoint. The pair of needles was connected to an EA device (Hanshi pain healing device, Hanshi-100A; Nanjing Jisheng Medical Technology Company, Nanjing, China). The current intensity was 1 mA, and the pulse frequency was 15 Hz, since studies by Han have shown that low frequencies yield better results; however, in future studies also 100 Hz will be investigated.

### 2.5. Procedure

**Figure 2:**
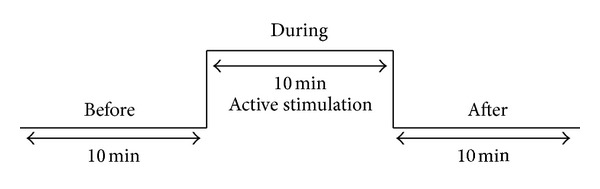
Experimental procedure for the different stimulation methods.

The measurement profile is shown in Figure 2. Three measurement periods were compared: one before, one during, and one after stimulation. This scheme was used for all three conditions (i.v. laser blood irradiation, i.st. laser acupuncture, and EA) in the same rat. The order of the stimulation methods was randomized, and the time between the separate measurements was at least 30 min.

### 2.6. Measurement Parameters

We registered electrocardiographic (ECG) and electroencephalographic (EEG) parameters using a biophysical amplifier AVB-10 (Nihon-Kohden, Japan). For the ECG, we evaluated heart rate (HR), heart rate variability (HRV), and the LF (low frequency)/HF (high frequency) ratio of HRV. EEG was registered directly on the brain; high cutoff frequency was 100 Hz, and the low cutoff frequency was 0.5 Hz. 

### 2.7. Statistical Analysis

The data were analyzed using one-way repeated measures analysis of variance (ANOVA) (SigmaPlot 12.0, Systat Software Inc., Chicago, USA). Post hoc analysis was performed using Holm-Sidak test. The level of significance was defined as *P* < 0.05.

## 3. Results

**Figure 3:**
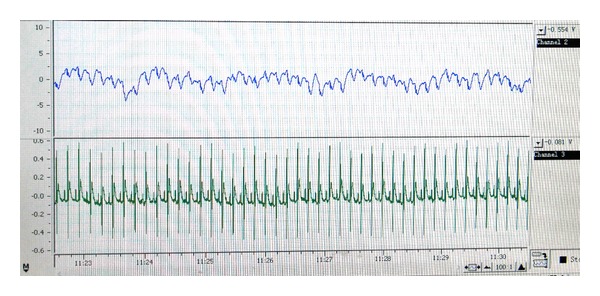
EEG (upper panel) and ECG (lower panel) raw data.

From a technical point of view, the data quality was very good in all 10 rats with a minimum of artifact.  Figure 3 shows an example of the raw data of the EEG and the ECG.

**Figure 4:**
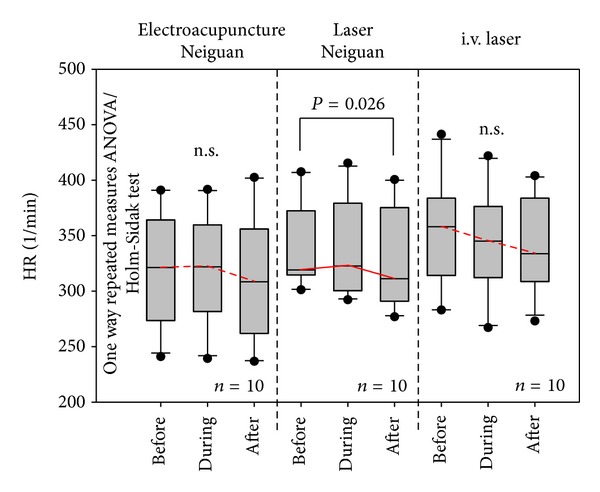
Box plots displaying the mean heart rate (HR) of the 10 rats. The ends of the boxes define the 25th and 75th percentiles with a line at the median and error bars defining the 10th and 90th percentiles. The different measurement phases (before, during, and after stimulation; compare with Figure 2) and different stimulation methods are indicated.

The analysis of the HR of all 10 rats is summarized in Figure 4. Note the significant (*P* = 0.026) decrease of HR after i.st. laser acupoint stimulation. It is also interesting that already in the phase during i.v. laser stimulation HR decreases, whereas HR decreased only in the phase after the two acupuncture stimulation methods. 

**Figure 5:**
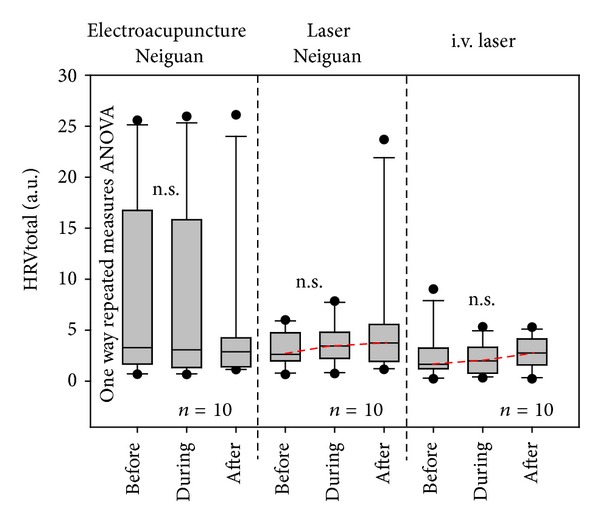
Changes in total heart rate variability (HRVtotal) before, during, and after the three stimulation procedures. For further explanation, compare with Figure 4.

In contrast to HR, total HRV increased slightly (insignificantly) during the two laser stimulation methods. No changes in total HRV were seen under EA (Figure 5). 

**Figure 6:**
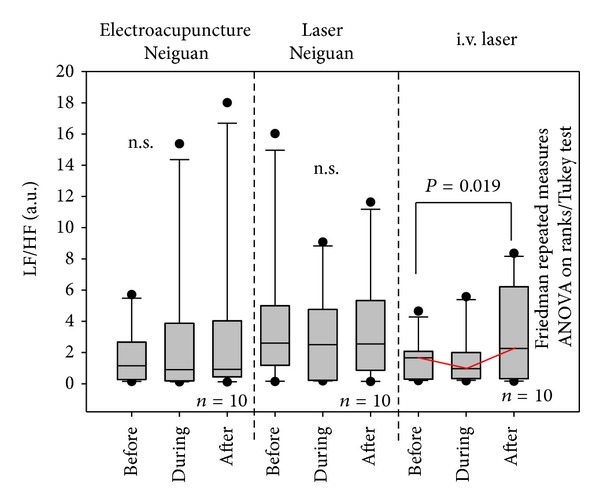
LF/HF of the 10 investigated rats. For further explanation, see Figure 4.

Figure 6 shows the changes of LF/HF. Significant changes were only found in the i.v. laser session.

**Figure 7:**
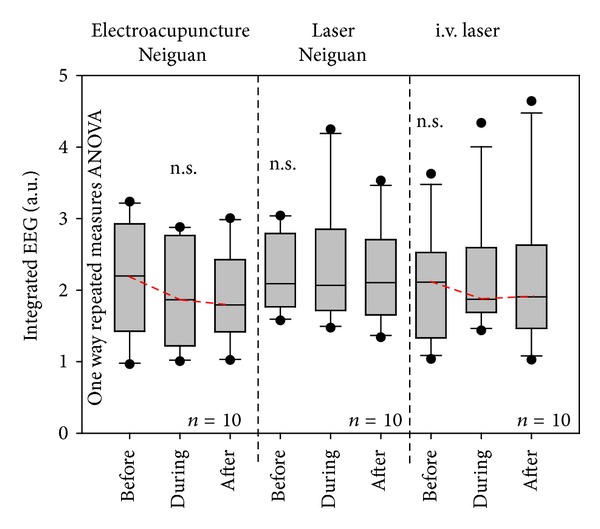
Statistical box plot analysis of the electrical rat brain activity. Note the (insignificant) decrease of the integrated EEG during EA and i.v. laser.

Analysis of the bioelectrical brain activity (EEG, Figure 7) revealed the following trends. There was a marked decrease in the slow frequencies during EA and i.v. laser stimulation, which, however, did not reach the level of statistical significance. Absolutely no changes were found in the i.st. laser acupuncture session. 

## 4. Discussion

Currently, there are about 150 publications in the database PubMed concerning i.v. laser irradiation of blood. However, only two papers on the topic “i.v. laser blood irradiation and EEG” can be found [14, 15]; both are in Russian language. One paper [14] describes i.v. laser therapy for circulatory encephalopathy, and the other [15] presents clinical data on therapeutic effects of i.v. laser blood irradiation in severe alcohol intoxication, complicated by alcohol coma. We did not find a publication in PubMed concerning “HRV and i.v. laser blood irradiation” or “electrocorticogram and i.v. laser blood irradiation.” However, this present publication is not the first one dealing with the topic i.v. laser irradiation in animal experiments, especially in rats. From 1988 to 2012, altogether 12 publications deal mainly with investigations of photodynamic therapy in rats [16–27]. Concerning humans, there are 86 publications on i.v. laser blood irradiation investigating different parameters; 80% of these studies are in Russian language. Since i.v. laser blood irradiation is already being often used in clinical practice, one of the goals of this study was to test its impact on neurovegetative parameters—a question hitherto unaddressed in research—in an animal experimental setting. 

In our study, we recorded for the first time electrocardiogram and electrocorticogram simultaneously and continuously during i.v. laser blood irradiation. We found a decrease in HR and also in the integrated corticogram and a small increase in HRV. Of course both EEG and HRV parameters are somewhat attenuated following the use of anesthetics, but since we are interested in changes in these parameters under otherwise steady-state conditions, this does not influence the conclusions that can be drawn from the results. Previous studies showed that some EEG components might be associated with the autonomic nervous modulation of the subject during positional change. It was suggested that there might be a mechanism located in the brain-stem which jointly controls both autonomic influences on heart rate and EEG activation [28]. In sleep apnea patients, the results have shown that EEG delta, sigma, and beta bands exhibited a strong correlation with cardiac HRV parameters at different sleep stages [29].

Our research group could show in previous studies that acupressure at the Yintang acupoint can induce EEG effects in humans [30]. Similar investigations with acupressure on the Extra 1 point by other authors found significantly reduced EEG spectral entropy in both genders, but LF/HF was affected only in females [31]. Manual stimulation on (Hegu) LI4 seems to lead to specific changes in alpha EEG frequency and in HRV parameters. A linear relationship between the HRV parameters and the alpha EEG band might point to a specific modulation of cerebral function by vegetative effects during acupuncture [32]. The relationship among specific sensations induced by acupuncture manipulation, effects on sympathetic and parasympathetic autonomic functions, and EEG changes has been investigated by Sakai et al. [33]. The authors demonstrated that acupuncture manipulation significantly decreased the LF spectral component of HRV and significantly reduced LF/HF, which is an index of sympathetic activity. They also found a significant negative correlation between changes in LF/HF and the number of specific acupuncture sensations reported and a significant positive correlation between HF of HRV and the number of acupuncture sensations. In addition, analyses of EEG data indicated that acupuncture manipulation nonspecifically increased power of all spectral bands except the gamma band. Furthermore, changes in HF (index of parasympathetic activity) and total power (overall activity of the autonomic nervous system) of HRV were positively correlated with changes in theta, alpha, and gamma power. These results are consistent with the suggestion that autonomic changes induced by manipulation inducing specific acupuncture sensations might be mediated through the central nervous system, especially through the forebrain as shown in EEG changes [33]. In our study, we only analyzed integrated EEG, and we did not perform detailed frequency analyses of EEG. This will be done in further studies with multichannel EEG (electrocorticogram, resp.) recordings.

As mentioned previously, the clinical use of i.v. laser blood irradiation is already widespread; yet there are only few studies dealing with basic research on this topic. Our present study is the first one comparing i.v. laser stimulation with acupuncture laser stimulation and comparing EA and laser acupuncture in rats. It is well known that the effectiveness of laser acupuncture depends upon dosage [34, 35]. We used a red laser (658 nm) with an output power of 50 mW, which results in a very high dosage. This dosage is also time dependent. In followup studies with this experimental rat model, it would be highly interesting to perform investigations with, for example, half the output power (25 mW), but twice the irradiation time (20 min instead of 10 min as used in this study). This is a very interesting topic which, to the best of our knowledge, has not been investigated in an experimental study design. 

## 5. Conclusions

The following conclusions can be drawn from the results of this animal experimental study.

- HR changes significantly during i.st. laser acupuncture stimulation of Neiguan in anesthetized rats.
- Total HRV increased insignificantly during i.v. and i.st. laser stimulation.
- LF/HF showed a significant increase only during i.v. laser blood irradiation, indicating an increase in sympathetic tone.
- Integrated cortical EEG (electrocorticogram) decreased insignificantly during EA and i.v. laser blood irradiation.
